# Seizure and Acute Vision Loss in a Filipino Lupus Patient: A Case of Posterior Reversible Encephalopathy Syndrome with Intraparenchymal Hemorrhage

**DOI:** 10.1155/2018/4238676

**Published:** 2018-12-09

**Authors:** Deonne Thaddeus V. Gauiran, Therese Eileen B. Lladoc-Natividad, Ida Ingrid I. Rocha, Bernadette Heizel Manapat-Reyes

**Affiliations:** ^1^Department of Medicine, UP-Philippine General Hospital, Manila, Philippines; ^2^Section of Rheumatology, Department of Medicine, UP-Philippine General Hospital, Manila, Philippines; ^3^Department of Neurosciences, UP-Philippine General Hospital, Manila, Philippines

## Abstract

Posterior reversible encephalopathy syndrome (PRES) is a rare and poorly understood neurologic condition that has been described in some patients with systemic lupus erythematosus (SLE). Intracerebral hemorrhage is a unique and atypical presentation of PRES and has been described only in a small number of patients with SLE. We present the case of a 33-year-old female, diagnosed with SLE and active nephritis, who was admitted for seizures. She had acute-onset headache, confusion, and bilateral vision loss associated with severe hypertension. CT scan revealed right occipital and parietal lobe hemorrhage. MRI showed vasogenic edema and hyperintense foci in bilateral cortical and subcortical regions of the occipital and posterior parietal lobes which are consistent with posterior reversible encephalopathy syndrome (PRES). Strict blood pressure control and medical ICP-lowering treatment were immediately instituted, while maintaining her on anticonvulsants, high-dose steroids, and mycophenolate mofetil. The patient was discharged with improvement in vision and resolution of headache. On follow-up, she had gained her premorbid visual acuity and reported no recurrence of headache or seizures. Despite its name, reversibility remains to be conditional in PRES. A high index of suspicion is important, especially among those who present with seizure, headache, and visual loss. Early diagnosis and timely initiation of therapy is recommended, as clinical symptoms are potentially reversible and delayed therapy may result in life-threatening complications, such as coma or death.

## 1. Introduction

Patients with systemic lupus erythematosus (SLE) present with neurologic complaints in 14–75% of cases [1]. The American College of Rheumatology established case definitions for 19 specific neuropsychiatric syndromes in SLE. These syndromes are categorized into those involving the central nervous system and those involving the peripheral nervous system. Neurologic symptoms vary from mild to severe; hence, there is the need for early diagnosis and timely initiation of treatment. Unfortunately, neuropsychiatric syndromes are often complicated by comorbid conditions and side effect of medications [1]. These factors pose challenges in the diagnosis and management of lupus patients presenting with neuropsychiatric complaints.

Posterior reversible encephalopathy syndrome (PRES) is a poorly understood neurologic condition that has been described in some patients with SLE. It has been considered by some to be more common than previously thought, and this may be due to underrecognition [2]. PRES, which is both a clinical entity and radiological entity, is characterized by acute-onset headache, vomiting, seizures, visual perception abnormalities, and parietooccipital white matter changes on magnetic resonance imaging (MRI) [3]. Among lupus patients, PRES has been identified especially among those with poorly controlled blood pressure, renal disease, or immunosuppressive therapy. In our review of the literature, we did not find any published reports on PRES, especially those presenting with intracranial hemorrhage, occurring among Filipino patients with SLE. In this paper, we present a successful management of a unique case of an SLE patient with a hemorrhagic presentation of PRES.

## 2. Case Report

A 33-year-old female was admitted at our institution for an episode of tonic seizure. She was diagnosed a month earlier with SLE after she presented with a photosensitive rash, arthritis, anemia, lymphocytopenia, albuminuria, hematuria, and positive antinuclear antibodies. At the outpatient clinic, she was initially started on prednisone at 1 mg/kg/day, hydroxychloroquine, and antihypertensive medications, to which she was strictly adherent. She claims to have had no previous episodes of headache, irritability, cognitive changes, or seizures, but reported poor sleep for the past week.

On initial evaluation, blood pressure was elevated at 160/100 mmHg. Other vital signs were normal. Pertinent physical examination findings included facial acneiform lesions, moon facies, and supraclavicular fullness. She also had grade 1 bipedal edema and left shoulder tenderness. The initial neurologic examination was unremarkable.

Initial laboratory work-up revealed microcytic, hypochromic anemia (hemoglobin of 99 g/L), and thrombocytopenia (platelet count of 100 × 10^9^/L). Direct and indirect Coombs test was positive. Serum creatinine and electrolytes were normal. Urinalysis showed +3 proteinuria, mild pyuria, and coarse granular casts. Total 24-hour urine protein was elevated at 4.47 grams. Assessment at this time was neuropsychiatric SLE with hematologic and renal lupus activity and poorly controlled hypertension. Prednisone was shifted to hydrocortisone 100 mg IV bolus every 8 hours, and combination of antihypertensive agents was continued. Mycophenolate mofetil at 2 g/day was also started.

On the second hospital day, the patient developed sudden-onset holocranial headache, confusion, and bilateral total loss of vision, which started out as blurring of peripheral visual fields a few hours prior. Blood pressure at this time was 190/110 mmHg. On examination, the patient was disoriented to time and place. Aside from bilateral total loss of vision, the rest of cranial nerve exam was normal. Muscle strength, deep tendon reflexes, and sensation were normal. Physical examination was unremarkable save for the presence of facial acneiform lesions, moon facies, and supraclavicular fullness which were already noted on admission. The patient was referred to neurology and ophthalmology for comanagement. Ophthalmology noted findings of bilateral optic atrophy and pathologic myopia. Neurology gave an initial assessment of posterior reversible encephalopathy syndrome (PRES) after noting similar neurologic findings.

**Figure 1:**
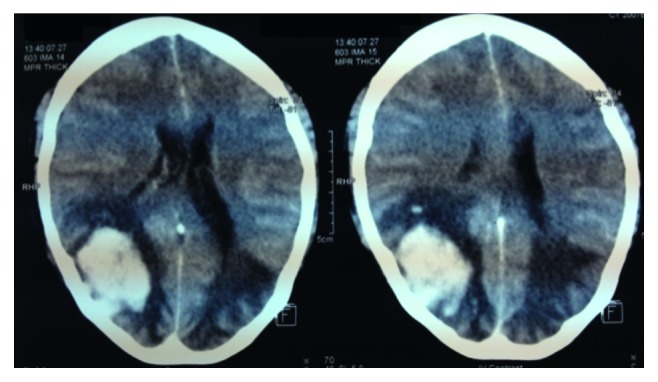
Contrast-enhanced cranial CT demonstrating a hemorrhagic focus in the right parietal and occipital lobes with possible secondary bilateral infarction due to compression of posterior circulation vessels, subfalcine herniation, beginning uncal herniation, and cerebral edema.

Contrast-enhanced cranial CT scan revealed acute parenchymal hemorrhage on the right occipital lobe amounting to 18.4 cc, with extension to the right parietal lobe, surrounding cerebral edema, subfalcine herniation, and beginning uncal herniation (Figure 1). Possible acute infarcts at the bilateral occipital and parietal lobes, which were initially thought to be due to compression of posterior circulation vessels by the hemorrhagic focus, were also considered. The patient was transferred to the intensive care unit for closer monitoring. Medical intracranial pressure- (ICP-) lowering treatment and strict blood pressure control were immediately instituted. Mannitol intravenous boluses, levetiracetam, and lactulose were started. Nicardipine drip was titrated to maintain systolic BP below 140 mmHg and mean arterial pressures between 110 and 120 mmHg. Oral antihypertensives were also carefully uptitrated. Neurosurgery was also called upon for comanagement. A ruptured arteriovenous malformation was entertained, and a 4-vessel angiogram was ordered. She was also worked up for antiphospholipid antibody syndrome (APAS). However, Kaolin clotting time (KCT) and diluted Russell viper venom time (DRVVT) levels were normal.

**Figure 2:**
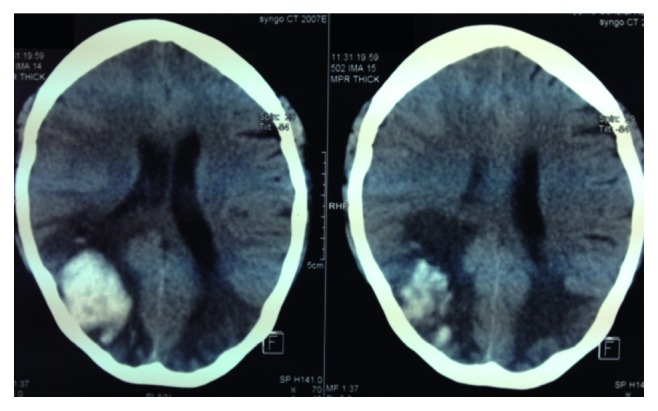
Plain cranial CT demonstrating a decrease in previously noted cerebral edema and midline shift.

**Figure 3:**
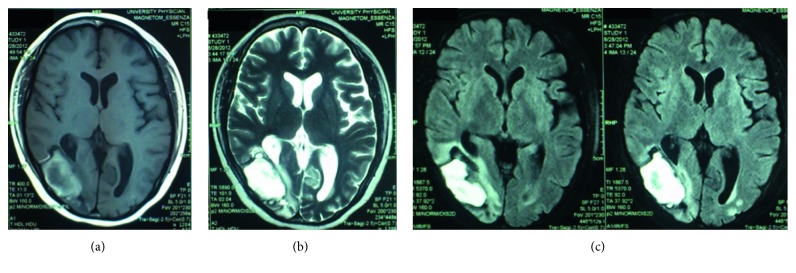
Plain cranial MRI images of the patient. (a) T1-weighted MR image demonstrating a hyperintense hematoma with hypointense core on the right occipital lobe, compressing the occipital horn of the right lateral ventricle, (b) T2-weighted MR image showing hyperintense signals of the same hematoma with surrounding vasogenic edema, and (c) FLAIR MR images demonstrating cortical-subcortical signal abnormalities on the right as well as left occipital lobes.

Further work-up included a 2D-echocardiogram which revealed an ejection fraction of 71%, eccentric hypertrophy with good wall motion and contractility, and a small pericardial effusion and no signs of myocarditis or rheologic stasis. In the days that followed, the patient reported gradual improvement in vision and headaches decreased in intensity and frequency. A repeat CT scan five days postictus revealed decreased cerebral edema especially on the left hemisphere and a relative decrease in midline shift (Figure 2). Four-vessel angiogram showed no evidence of arteriovenous malformation and only showed poor parenchymal staining in the right parietal region due to the hematoma. Cranial MRI (done without contrast due to financial constraints) at seven days postictus revealed a 4.6 × 4.3 × 2.5 cm parenchymal hematoma in the right parietooccipital area with a volume of 26 cc, compressing the occipital horn of the right lateral ventricle (Figure 3). The hematoma was peripherally hyperintense and centrally hypointense on T1-weighted images, with surrounding areas of encephalomalacia. Patchy foci of T2 FLAIR hyperintensities are seen in the cortical and subcortical regions of the occipital and posterior parietal lobes bilaterally. These findings are consistent with PRES.

On follow-up six weeks postictus, the patient had gained her premorbid visual capacity and reported no recurrence of severe headache or seizures. She continues to be on prednisone, mycophenolate mofetil, hydroxychloroquine, amlodipine, losartan, carvedilol, and levetiracetam.

## 3. Discussion

Posterior reversible encephalopathy syndrome (PRES) is characterized by a constellation of symptoms including headache, altered mental status, vision loss, seizures, and coma. It was first described by Hinchey et al. in 1996 and was named reversible posterior leukoencephalopathy (RPLS) [3]. In 2000, Casey et al. proposed the term posterior reversible encephalopathy syndrome to emphasize the common involvement of both the grey and white matter [4]. Since then, PRES had been associated with various conditions such as hypertensive crises, preeclampsia, eclampsia, immunosuppression, transplantation, connective tissue disorders, uremia, and infections.

The mechanism of PRES is not yet well elucidated. It is generally thought to be multifactorial and related to endothelial cell dysfunction. This leads to blood-brain barrier leakage and subsequent cortical and subcortical, predominantly vasogenic, and occasional cytotoxic edema [2]. In SLE, autoimmune and ischemic (vasculitic and thrombotic) complications may likewise bring about endothelial dysfunction [2]. Additionally, cytotoxic drugs used in SLE may also lead to endothelial damage [2]. Nephritis, a common life-threatening presentation of SLE, can also promote vasogenic edema through fluid retention and hypertension [2]. Hypertension, active lupus nephritis, and current treatment with corticosteroids are important factors which may explain the development of PRES in our patient.

In 2007, Karoui et al. reviewed 31 cases of SLE patients presenting with PRES. In this review, it was noted that most of the cases had a history of lupus nephritis and most had recently received corticosteroids and cyclophosphamide [2]. Only two of thirty-one cases had normal blood pressure. Gatla et al. in 2013 described another cohort of five patients with SLE presenting with PRES, all of which had hypertension and moderate-to-severe SLEDAI disease activity [5]. The largest cohort of 103 SLE patients with PRES was reported in 2013 by Shaharir et al. [6]. Majority of the patients were females and Asians with a mean age of 26.3 +/− 8.8 years [6]. Our patient also falls within the same age range. The three most common presentations were seizures, headache, and sensorial changes, all of which were seen in our patient. Also consistent with our case, PRES was noted to be highly associated with active SLE, hypertension, and nephritis [6]. Our patient was diagnosed with SLE a month prior, with recent initiation of high-dose steroids. Although a kidney biopsy was not done (due to financial constraints), the nephritis is probably proliferative, based on the presence of hypertension and active urine sediments on routine urinalysis.

Imaging findings and reversibility are important in distinguishing PRES from differential diagnoses such as bilateral ischemic strokes in the posterior cerebral artery territory, central venous sinus thrombosis, demyelinating disorders, lupus encephalitis, cerebral vasculitis, and infectious or metabolic encephalopathy which are all seen in patients with SLE.

Imaging in PRES typically demonstrates focal areas of symmetric hemispheric edema usually involving the parietal and occipital lobes and occasionally, the frontal lobes, inferior temporal-occipital junction, and the cerebellum [7, 8]. This pattern of involvement may be explained by differences in autonomic innervation between the anterior and posterior regions of the brain, with the posterior region possessing poorer sympathetic tone, predisposing this region to develop edema [2, 5]. Our patient presented with hemorrhage on the right occipital lobe, extending to the right parietal lobe with surrounding edema on her initial cranial CT scan. However, the presence of vasogenic edema even on the contralateral side strengthened our consideration of PRES in this case. The cortical hyperintensities on the occipital and posterior parietal lobes, absence of typical pattern on cerebral angiography, and the lack of signal or flow voids on MRI made the diagnosis of AVM less likely.

Cerebral hemorrhage is an uncommon finding in PRES and has been reported in only 5 to 17% of patients with PRES [8–10]. Likewise, intracranial hemorrhage is not commonly seen in SLE-associated PRES [5, 6, 11]. In a review done by Hefzy et al. in 2009, two (16.7%) out of twelve patients with autoimmune disease (SLE or scleroderma) had minute petechial hemorrhages. One of them also demonstrated a coexisting intracerebral hematoma [11]. In a larger review by Shaharir et al. in 2013, only six (8.6%) out of 70 patients with SLE-associated PRES showed hemorrhagic radiographic changes [6]. The mechanism for the occurrence of cerebral hemorrhage in SLE is not yet well elucidated. It is thought to occur due to small vessel rupture and/or postischemic reperfusion injury [6, 7, 11]. Anticoagulant treatment was noted to increase the risk of hemorrhage, whereas blood pressure levels may have no influence on the risk of bleeding [11]. Our patient has coexisting active nephritis, anemia, and mild thrombocytopenia which may have also possibly predisposed the patient to have intracranial hemorrhage.

Despite the moniker, PRES may not be a fully reversible condition. Cerebral infarction, subarachnoid hemorrhage, coma, or death has been described in PRES [2, 12, 13]. Among those who presented with intracranial hemorrhage, 16 (70%) of 23 patients had complete neurologic recovery [11]. A mortality rate of 26% was reported with an average survival of 17 days (range: 1–64 days) [11]. Furthermore, the review of Shaharir et al. identified intracranial hemorrhage (OR 14, 1.1–187.2, *p*=0.04) and brainstem involvement (OR 10.9, 1.3–90.6, *p*=0.003) as predictors of poor outcome [6]. Fortunately, despite the occurrence of intraparenchymal hemorrhage, our patient did not develop any motor or sensory deficits and long-term vision loss.

The management of PRES is largely supportive [5]. Treatment involves adequate blood pressure control, with close titration of parenteral antihypertensives to prevent cerebral hypoperfusion. Hemodialysis may be warranted for those presenting with severe fluid retention [2]. Regardless of etiology, acute seizures should be controlled with antiepileptic agents [2]. Among patients whose PRES are probably caused by immunosuppressive therapy, temporary dose adjustment or withdrawal of inciting drugs may be warranted until signs of PRES abate [2]. Treatment directed toward the underlying cause is also important in the management of PRES [5]. In patients with active lupus nephritis, corticosteroids and/or cytotoxic agents may be needed to control the disease activity. Careful monitoring is especially necessary since these medications may worsen PRES. In our patient, levetiracetam was started for seizure management. Mannitol was initiated for intracranial pressure lowering, and nicardipine infusion was started for blood pressure control. Combination oral-hypertensives were also uptitrated to affect better blood pressure control. Hydrocortisone, mycophenolate mofetil, and hydroxychloroquine were maintained to manage the lupus disease activity. With these medications, the patient was noted to improve clinically, with control of seizures and resolution of headache and visual deficits. PRES should then be promptly recognized and treated as clinical symptoms are potentially reversible with timely management.

## 4. Conclusion

We have presented a case of a patient with SLE who developed signs, symptoms, and neuroimaging findings consistent with an atypical neuroradiologic presentation of PRES, which is of intraparenchymal hemorrhage. After early initiation of blood pressure and seizure control and medical ICP-lowering treatment, further neurologic decline was halted and visual deficits improved. This case underscores the importance of early recognition and treatment of a relatively unique and potentially reversible condition which may present alongside the diverse neurologic manifestations of SLE.
